# Aspectos técnicos y clínicos de la prueba cruzada de histocompatibilidad en el trasplante de órganos solidos

**DOI:** 10.7705/biomedica.6255

**Published:** 2022-06-01

**Authors:** Ana María Arrunátegui, Daniel S. Ramon, Luz Marina Viola, Linda G. Olsen, Andrés Jaramillo

**Affiliations:** 1 Departamento de Patología y Medicina de Laboratorio, Fundación Valle del Lili, Cali, Colombia Fundación Valle del Lili Cali Colombia; 2 Department of Laboratory Medicine and Pathology, Mayo Clinic, Phoenix, AZ, United States of America Mayo Clinic Phoenix AZ United States of America

**Keywords:** trasplante de órganos, antígenos HLA, histocompatibilidad, pruebas inmunológicas de citotoxicidad, citometría de flujo, Organ transplantation, HLA antigens, histocompatibility, cytotoxicity tests, immunologic, flow cytometry

## Abstract

La presencia de anticuerpos dirigidos contra los antígenos leucocitarios humanos (*Human Leukocyte Antigens*, HLA) que se expresan en las células del donante, es uno de los factores de riesgo más importantes asociados con las complicaciones clínicas después del trasplante. La prueba cruzada es una de las pruebas de histocompatibilidad más eficaces para la detección de anticuerpos específicos contra el donante en los receptores de injertos. En los primeros métodos de la prueba cruzada, se utilizaba la citotoxicidad dependiente del complemento, que es útil para detectar dichos anticuerpos responsables del rechazo hiperagudo del injerto, pero carece de la sensibilidad adecuada. Por ello, se desarrollaron métodos de pruebas cruzadas más sensibles, entre ellas, la prueba cruzada por citometría de flujo que hoy se considera el método preferido.

En este artículo se revisa la evolución de la prueba cruzada y los factores más importantes que deben tenerse en cuenta al realizarla y al interpretar los resultados de esta prueba fundamental para la supervivencia a largo plazo del injerto.

## Abstract (en)

The presence of antibodies directed against human leukocyte antigens (HLA) expressed on donor cells is a significant risk factor for serious clinical complications after transplantation. The crossmatch assay is one of the most important tests available for the detection of donor-specific antibodies in potential allograft recipients. Early crossmatch methods utilized complement-dependent cytotoxicity, which is useful for detecting the donor-specific anti- HLA antibodies responsible for hyperacute allograft rejection but lacks adequate sensitivity. Consequently, more sensitive crossmatch methods have been developed, ultimately leading to the flow cytometry crossmatch as the currently preferred methodology.

Herein, we review the evolution of the crossmatch assay and the most important factors to consider when performing and interpreting the results of this fundamental assay for ensuring the long-term survival of the transplanted organ.

Dos de las barreras inmunológicas más importantes en el trasplante de órganos, son la incompatibilidad del grupo sanguíneo ABO y la presencia de anticuerpos dirigidos contra los antígenos leucocitarios humanos (*Human Leukocyte Antigens*, HLA) en los receptores potenciales de injertos. La presencia de anticuerpos específicos contra el donante es un importante factor de riesgo para complicaciones clínicas después del trasplante. Dependiendo del nivel de los anticuerpos específicos contra el donante, el impacto clínico puede variar desde eventos menores, controlados con inmunosupresión estándar, hasta complicaciones graves como el rechazo hiperagudo del injerto que conducen a perderlo ^1^^-^^5^.

Independientemente del grado de compatibilidad del HLA, la prueba de histocompatibilidad más importante que se utiliza en el laboratorio es la cruzada. En el método original, se utiliza la citotoxicidad dependiente del complemento para la detección de anticuerpos específicos contra el donante en el suero del paciente. Si bien este método es adecuado para detectar la mayoría de estos anticuerpos responsables del rechazo hiperagudo del injerto, no detecta niveles más bajos que conducen al rechazo acelerado del injerto y a su pérdida temprana. Como consecuencia de lo anterior, se desarrollaron métodos de prueba cruzada más sensibles, lo que condujo finalmente a adoptar la prueba cruzada por citometría de flujo como el método preferido actualmente.

En esta revisión, se examina la evolución de la prueba cruzada y los factores más importantes que deben tenerse en cuenta al realizarla y al interpretar los resultados para garantizar la supervivencia a largo plazo del injerto ^6^^-^^11^.

## Prueba cruzada por citotoxicidad dependiente del complemento

La prueba cruzada por citotoxicidad dependiente del complemento (*Complement-Dependent Microcytotoxicity Crossmatch*, CDCXM) se introdujo en 1969 en un artículo histórico que demostró por primera vez que los receptores de trasplantes renales con una prueba cruzada positiva en el momento del trasplante tenían una tasa significativamente mayor de rechazo hiperagudo ^12^. Por lo tanto, la CDCXM se convirtió inmediatamente en una prueba de referencia obligatoria para la detección de anticuerpos específicos contra el donante antes del trasplante. Después de establecer la obligatoriedad de un resultado negativo en dicha prueba, se observó una reducción significativa en la tasa de rechazo hiperagudo del injerto ^12^^,^^13^.

La CDCXM es una prueba biológica que requiere la unión de anticuerpos, la activación del complemento y la lisis celular, para indicar una reacción positiva. Los linfocitos del donante se incuban primero con el suero del receptor potencial, después de adicionar suero de conejo como fuente de complemento. Si los anticuerpos en el suero del receptor potencial reconocen HLA expresados por las células del donante, se inicia la activación de la cascada clásica del complemento, lo que lleva a la formación del complejo de ataque a la membrana celular y a la desintegración celular. El grado de lisis celular se determina por la inclusión de colorantes supravitales, como el azul de tripán o la eosina, o de colorantes fluorescentes que se unen al ácido desoxirribonucleico, como el bromuro de etidio. Los resultados se interpretan utilizando un sistema de clasificación estandarizado basado en el porcentaje de células desintegradas ^14^^-^^16^. Aunque esto significa una mejoría frente a la ausencia de una prueba de histocompatibilidad, la CDCXM tiene una tasa significativamente alta de resultados falsos negativos (baja sensibilidad) y falsos positivos (baja especificidad), es decir, carece de la sensibilidad necesaria para detectar todos los anticuerpos específicos contra el donante relevantes y excluye de manera inapropiada los receptores potenciales de injertos debido a los falsos positivos ^17^^-^^19^.

En un intento por resolver los problemas de sensibilidad y especificidad de la CDCXM, se introdujeron varias modificaciones en el método original, entre otras, lavados adicionales, incubaciones prolongadas y la adición de antiglobulina humana. En este sentido, la modificación de la CDCXM más utilizada es la adición de antiglobulina humana (*Anti-Human Globulin*, AHG) para aumentar la capacidad de activación del complemento de la muestra ^20^. Esta inmunoglobulina es un anticuerpo activador del complemento dirigido contra la inmunoglobulina G (IgG) humana, que se agrega como un segundo paso y se une a los anticuerpos unidos a las células blanco, por lo que aumenta la densidad de anticuerpos, la probabilidad de activación del complemento y la sensibilidad de la prueba.

Por lo tanto, la CDCXM con adición de AHG (AHG-CDCXM) se impuso rápidamente frente a la CDCXM y desempeñó un papel importante en la reducción de la tasa de rechazo acelerado del injerto al detectar niveles más bajos de anticuerpos específicos contra el donante que, por su baja concentración, no activan el complemento, lo que se conoce como anticuerpos con citotoxicidad negativa y adsorción positiva (*CytotoxicNegative, Absorption-Positive*, CYNAP) ^17^^,^^18^^,^^21^. Los anticuerpos específicos detectados por la AHG-CDCXM pero no por la CDCXM, están asociados con una tasa mayor de pérdida temprana del injerto, en comparación con la de pacientes con una AHG-CDCXM negativa ^19^^,^^21^^,^^22^.

Cabe señalar que tanto la CDCXM como la AHG-CDCXM pueden detectar anticuerpos de clase IgG e inmunoglobulina M (IgM). Así, aunque los anticuerpos de clase IgM no se consideran clínicamente significativos, pueden interferir con la detección de anticuerpos de clase IgG. Por lo tanto, una práctica común para aumentar la especificidad de la prueba es el tratamiento previo del suero del paciente con agentes reductores, como el ditiotreitol, para dividir la IgM pentamérica en monómeros, haciéndola incapaz de activar el complemento ^21^. Otra práctica común para aumentar la especificidad de la prueba es el tratamiento previo del suero del paciente con altas temperaturas, usualmente 56 °C durante 30 minutos, para inactivar el complemento ^23^.

En conclusión, la CDCXM no puede detectar los anticuerpos específicos contra el donante que no activan el complemento o que se encuentran en concentraciones inferiores al umbral necesario para su activación. Además, la CDCXM muestra una sensibilidad significativamente baja para la detección de anticuerpos específicos contra el donante de clase II, lo cual no mejora con la adición de AHG debido a la unión inespecífica de esta a los receptores del fragmento cristalizable de la inmunoglobulina (*Fc Receptor*, FcR) en los linfocitos B ^24^.

Otra desventaja de la CDCXM es la dificultad de obtener un número suficiente de células viables para la prueba, especialmente linfocitos B, lo que hace muy difícil la interpretación de resultados con este tipo de células.

Otras desventajas de la CDCXM incluyen la muerte celular inespecífica que conduce a resultados falsos positivos y la presencia de factores inhibidores del complemento en el suero del paciente que conducen a resultados falsos negativos. Todos estos factores pueden interferir en la eficacia de la prueba, lo que da lugar a una variabilidad significativamente grande entre laboratorios ^25^. Aunque la AHG-CDCXM representa una mejoría con respecto a la CDCXM, esta presenta los mismos problemas intrínsecos de los sistemas de detección de anticuerpos dependientes de la activación del complemento ^24^^,^^25^.

## Prueba cruzada por citometría de flujo

Si bien el trabajo original de Patel, *et al*. ^12^, y varios estudios posteriores demostraron una asociación entre una CDCXM positiva y los rechazos hiperagudos y acelerados del injerto, un número significativo de pacientes con una CDCXM negativa también experimentaban este tipo de rechazo ^17^^-^^19^^,^^21^^,^^22^^,^^26^. Con la introducción en 1983 de la prueba cruzada por citometría de flujo (*Flow Cytometry Crossmatch*, FCXM), se logró una mejoría significativa de la sensibilidad y la especificidad de la prueba ^27^.

Este y otros estudios posteriores demostraron que la FCXM puede detectar niveles bajos de anticuerpos específicos contra el donante que la CDCXM no logra rastrear ^27^^-^^30^. Esto es particularmente importante para los receptores de trasplantes renales con una CDCXM o una AHG-CDCXM negativas, pero una FCXM positiva, pues tienen una probabilidad mayor de experimentar episodios de rechazo hiperagudo o acelerado del injerto y su pérdida temprana ^31^^-^^34^. Por lo tanto, la FCXM ha reemplazado rápidamente a la CDCXM y la AHG-CDCXM como la prueba de referencia obligatoria para detectar tales anticuerpos específicos contra el donante antes del trasplante.

La FCXM se hace incubando las células del donante con el suero del paciente. Si se encuentran anticuerpos específicos contra el donante en el suero del paciente, estos se unen a los HLA en las células del donante. Después de un paso de lavado para remover anticuerpos no específicos contra el donante y otros componentes del suero, la evaluación de la unión del anticuerpos específicos contra el donante se evalúa mediante la adición de un anticuerpo contra la IgG humana conjugado con isotiocianato de fluoresceína para detectar los anticuerpos específicos contra el donante unidos a las células. Así, la intensidad de la fluorescencia emitida por el isotiocianato de fluoresceína es proporcional a la cantidad de anticuerpos específicos contra el donante unidos a las células.

En una modificación posterior, se introdujo la FCXM de tres colores para permitir la evaluación simultánea de las poblaciones de linfocitos T y B sin necesidad de separarlas físicamente ^30^. Esta técnica incorpora una incubación adicional con un anticuerpo monoclonal contra el CD3 conjugado con el complejo proteico de peridinina-clorofila y un anticuerpo monoclonal contra el CD19 conjugado con ficoeritrina para identificar las poblaciones de linfocitos T y B, respectivamente. Así, la FCXM de tres colores permite la detección simultánea de los anticuerpos específicos contra el donante que reaccionan con las poblaciones de linfocitos T y B, excluyendo las células mononucleares no linfoides del análisis.

La interpretación de la FCXM se hace comparando la intensidad de la fluorescencia de las células incubadas con el suero del paciente y la intensidad de la fluorescencia de las células incubadas con un suero de control negativo. Los resultados se reportan comúnmente como la diferencia entre las medianas de los canales de fluorescencia del suero del paciente y el control negativo (*Median Channel Shift*, MCS). La interpretación de la FCXM es más objetiva que la interpretación visual utilizada en la CDCXM y tiene la ventaja adicional de ser semicuantitativa en el sentido de que el MCS es proporcional a la cantidad de anticuerpos específicos contra el donante unidos a las células ^6^^,^^7^^,^^10^^,^^11^^,^^28^^,^^29^^,^^35^^,^^36^.

Si bien la FCXM es un método con excelente sensibilidad y especificidad, también comparte varios de los problemas asociados a las pruebas celulares ^24^^,^^37^^,^^38^. Entre los problemas más importantes, podemos mencionar la unión inespecífica de las inmunoglobulinas a los FcR, los autoanticuerpos no HLA y el uso de anticuerpos terapéuticos para inducción de la inmunosupresión, como rituximab (anti-CD20), daclizumab (anti-CD25), alemtuzumab (anti-CD52) y globulina antitimocítica, que dan lugar a resultados falsos positivos ^39^^-^^43^.

## Expresión y distribución variable del antígeno leucocitario humano

Es importante mencionar que los linfocitos T expresan HLA de clase I, mientras que los linfocitos B expresan HLA de clase II y niveles más altos de HLA de clase I ^24^^,^^44^^-^^48^. Debido a esta expresión y distribución diferencial del HLA en los linfocitos T y B, la FCXM de tres colores proporciona información adicional sobre la reactividad del anticuerpos específicos contra el donante (HLA de clase I *versus* HLA de clase II) (cuadro 1). También, se ha observado que la expresión varía entre los diferentes loci del HLA, siendo más alta para los HLA-A, B y DRB1 que para los HLA-C, DRB3/B4/B5, DQB1 y DPB1 ^44^^,^^45^^,^^47^^,^^49^.

**Cuadro 1:** Interpretación de la prueba cruzada por citometría de flujo

Linfocitos T	Linfocitos B	Anticuerpos específicos contra el donante
Negativo	Negativo	Negativo o bajo nivel
Positivo	Positivo	HLA de clase I o HLA de clase I y II
Negativo	Positivo	HLA de clase II
Positivo	Negativo	HLA-C*

* Observado en algunos casos

La expresión del HLA varía igualmente entre diferentes alelos del mismo locus ^50^^-^^52^. Además, varios estudios han demostrado que el HLA-C tiene un nivel de expresión más alto en linfocitos T comparado con su expresión en linfocitos B ^53^^-^^55^. Esto produce, en algunos casos, una FCXM con linfocitos T positivos y linfocitos B negativos (cuadro 1) ^53^^-^^55^.

Otros estudios han demostrado una variabilidad significativa de la expresión del HLA en linfocitos de diferentes tipos de donantes (vivos o fallecidos) y diferentes tejidos (sangre periférica, ganglios linfáticos o bazo) ^56^. En estos estudios se demostró, asimismo, que la variabilidad en la expresión del HLA tiene un impacto significativo en los resultados de la FCXM. La expresión del HLA de clase I es similar en los linfocitos B de sangre periférica de donantes vivos y en el bazo y los ganglios linfáticos de donantes fallecidos, pero significativamente menor en los linfocitos B de sangre periférica de estos donantes. Por el contrario, la expresión del HLA de clase I en los linfocitos T y la del HLA de clase II en los linfocitos B, es significativamente mayor en sangre periférica de donantes vivos que en todos los tejidos de donantes fallecidos.

Cabe destacar que, entre los tejidos de donantes fallecidos, el bazo proporciona la mayor expresión de HLA de clase I en los linfocitos T y de HLA de clase II en los linfocitos B. Más importante aún, las diferencias de la expresión del HLA en linfocitos aislados de diferentes tejidos de un mismo donante influyen significativamente en los resultados de la FCXM. La expresión del HLA no se analiza rutinariamente en el momento de realizar la FCXM, pero puede impactar significativamente en su interpretación. También, es pertinente considerar que la expresión de los HLA se ve afectada por factores extrínsecos como edad, infecciones, enfermedades y medicamentos ^57^^-^^60^.

## Preparación de las células blanco

Las fuentes más comunes de células blanco para la FCXM son las células mononucleares derivadas de sangre periférica, ganglios linfáticos o bazo. La centrifugación en gradiente de densidad todavía se utiliza comúnmente para el aislamiento de células mononucleares. Sin embargo, dependiendo de la calidad de la muestra, se observa una gran variación en la pureza y el número de células mononucleares entre diferentes donantes cuando se usa este procedimiento ^10^^,^^11^^,^^61^^,^^62^.

Así, la pureza de la población linfoide en preparaciones de células mononucleares aisladas por centrifugación en gradiente de densidad, es relativamente baja y muy variable. En este sentido, en un estudio realizado en nuestro laboratorio (Mayo Clinic, Phoenix, AZ, USA), la selección negativa produjo una pureza de la población linfoide de 96,2 ± 3,7 % en sangre periférica y de 91,9 ± 3,7 % en el bazo. Por el contrario, la centrifugación en gradiente de densidad produjo una pureza de la población linfoide de 57,1 ± 9,2 % en sangre periférica y de 10,1 ± 0,6 % en el bazo ^10^^,^^11^^,^^63^.

Por ello, la presencia de células no linfoides, que también expresan moléculas de HLA, en la preparación de células mononucleares puede tener un efecto considerable en los resultados de la FCXM. Si el suero del paciente contiene baja concentración de anticuerpos específicos contra el donante, parte de este se une a las células no linfoides reduciendo la cantidad de los disponibles para la población linfoide, lo que puede arrojar resultados falsos negativos ^10^^,^^11^^,^^61^^,^^62^. Los resultados resumidos en el cuadro 2 demuestran que la FCXM realizada con linfocitos aislados por medio de selección negativa, presenta una sensibilidad significativamente mayor que la que se hace con linfocitos aislados mediante centrifugación en gradiente de densidad ^10^^,^^11^^,^^63^.

**Cuadro 2:** Características de rendimiento del método de centrifugación en gradiente de densidad comparado con el método de selección negativa

	FCXM de linfocitos T (n=20)		FCXM de linfocitos B (n=20)	
	Centrifugación en gradiente de densidad		Centrifugación en gradiente de densidad	
	Positivo	Negativo	Positivo	Negativo
Selección negativa				
Positivo	13	4	15	4
Negativo	0	3	0	1
Sensibilidad (IC_95%_)	76,4 % (50,1 - 93,2 %)		79 % (54,4 - 94 %)	
Especificidad (IC)	100 % (29,2 - 100 %)		100 % (2,5 - 100 %)	
VPP (IC_95%_)	100 % (75,3 - 100 %)		100 % (78,2 - 100 %)	
VPN (IC_95%_)	42,9 % (9,8 - 81,6 %)		20 % (0,5 - 71,6 %)	
Valor de p	0,03		0,25	

FCXM: prueba cruzada por citometría de flujo; IC: intervalo de confianza; VPP: valor predictivo positivo; VPN: valor predictivo negativo

**Figura 1:**
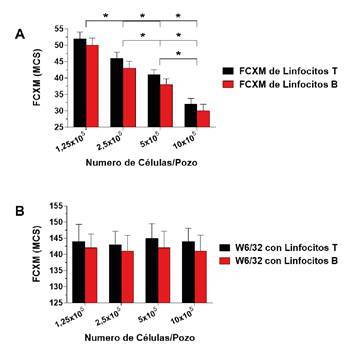
Correlación inversa entre el número de células y la reactividad de la prueba cruzada por citometría de flujo. La FCXM se hizo con un suero que contenía niveles bajos de anticuerpos especificos contra el donante con un número variable de células (1,25 x 10^5^ - 10 x 10^5^ células/pozo) (n=5). **A.** La evaluación cualitativa de la expresión del HLA se determinó por medio de incubación de las células con un anticuerpo monoclonal anti-HLA de clase I (W6/32) conjugado con isotiocianato de fluoresceína (5 μg/ml) (n=5). **B.** Los resultados se expresan como la media ± desviación estándar. La intensidad de la fluorescencia de la FCXM se captó como datos lineales en la escala de 0-256 canales.

Una de las variables más importantes al realizar la FCXM es la relación entre células y suero, pues, al aumentar el volumen de suero o reducir el número de células, aumenta la sensibilidad y, al reducir el volumen de suero o aumentar el número de células, esta disminuye ^10^^,^^11^^,^^61^^-^^63^. En la figura 1A se presentan los resultados de una FCXM en un suero con bajos niveles de anticuerpos específicos contra el donante y cuatro diluciones seriadas de una muestra con una concentración de 10 x 10^5^ células/pozo en los que se observa una disminución significativa en la intensidad de fluorescencia a medida que aumenta el número de células ^10^^,^^11^^,^^63^. Estos resultados demuestran que el número de células es inversamente proporcional a la reactividad de la FCXM y puede tener un importante efecto en su resultado, especialmente en los pacientes con bajos niveles de anticuerpos específicos contra el donante.

Cabe destacar que el mismo experimento realizado con una concentración óptima del anticuerpo monoclonal anti-HLA de clase I (W6/32, 5 μg/ml) no se vio afectado por el número de células (figura 1B) ^10^^,^^11^^,^^63^, lo que puede explicarse por la gran afinidad y la mayor concentración de saturación del anticuerpo monoclonal utilizado, que rara vez se observa en el suero de un paciente.

La FCXM es una prueba desarrollada en el laboratorio, por lo que el número de células y el volumen de suero utilizados en ella varían significativamente entre laboratorios. La mayoría de los laboratorios utilizan un número de células entre 3 x 10^5^ y 5 x 10^5^ por pozo y un volumen de suero entre 20 y 50 μl ^10^^,^^11^^,^^62^. Generalmente, se recomienda un número de células que no exceda 5 x 10^5^ por pozo y un volumen de suero que no sea menor de 20 μl ^10^^,^^11^^,^^62^. Esta heterogeneidad explica la gran variabilidad de los resultados de la FCXM entre laboratorios ^24^^,^^37^^,^^38^.

## Efecto de la pronasa en la prueba cruzada por citometría de flujo

Como ya se ha mencionado, los linfocitos T expresan solo el HLA de clase I, en tanto que los linfocitos B expresan HLA de clase I y de clase II ^24^^,^^44^^,^^45^^,^^47^. Por lo tanto, la FCXM de linfocitos B se considera una prueba más sensible para evaluar a los potenciales receptores de injertos ^24^. Sin embargo, la unión inespecífica de las inmunoglobulinas a los FcR y la expresión de receptores de complemento en los linfocitos B, producen una tasa alta de resultados falsos positivos ^24^. Esto ha dado lugar a importantes controversias sobre si la FCXM de linfocitos B tiene algún valor clínico ^24^.

Para superar este problema, Lobo, *et al*., y Vaidya, *et al*., utilizaron células pretratadas con pronasa para aumentar la especificidad de esta prueba cruzada con citometría de flujo ^64^^-^^68^. La pronasa es una mezcla de enzimas proteolíticas inespecíficas que se utiliza para remover los FcR de los linfocitos B, eliminando así la unión inespecífica de las inmunoglobulinas. Sus hallazgos subrayaron la importancia de mejorar la especificidad de la FCXM de linfocitos B, así como el valor de una mejor sensibilidad para la detección de los anticuerpos específicos contra el donante de clase II y los bajos niveles de los de clase I, que solo se detectan en los linfocitos B.

Sin embargo, el tratamiento con la pronasa no está exento de problemas. En varios estudios se ha demostrado que la incubación prolongada o el tratamiento con una alta concentración de pronasa (2 mg/ml) puede afectar la conformación de la molécula del HLA al destruir epítopos alogénicos y exponer epítopos crípticos que afectan el resultado de la FCXM ^69^. También, se demostró que el tratamiento con pronasa (1 mg/ml) aumenta significativamente la reactividad de la FCXM de linfocitos T, lo que indica que puede dar lugar a resultados falsos positivos. En este sentido, Park, et al., demostraron que el tratamiento con pronasa (1 mg/ml) induce un 13,3 % de resultados falsos positivos con la FCXM de linfocitos T ^70^. Además, en un estudio de nuestro laboratorio (Mayo Clinic, Phoenix, Arizona, USA), se demostró que el tratamiento con pronasa (1 mg/ml) induce resultados falsos positivos de la FCXM de linfocitos T en pacientes infectados con el virus de la inmunodeficiencia humana (*Human Immunodeficiency Virus*, HIV) ^42^ y que la reactividad de la prueba de linfocitos T tratados con pronasa fue inducida por autoanticuerpos no HLA ^42^.

En general, estos datos indican que el tratamiento con pronasa induce resultados falsos positivos en la FCXM de linfocitos T en ciertos grupos de pacientes. Por lo tanto, los laboratorios que utilizan pronasa en esta prueba deben examinar continuamente la tasa de resultados falsos positivos de la FCXM de linfocitos T y considerar la posibilidad de usar células sin tratamiento de pronasa para pacientes infectados con el HIV para evitar la exclusión inadecuada de un órgano compatible debido a un resultado falso positivo en la FCXM de linfocitos T en este grupo de pacientes.

## Anticuerpo secundario contra la inmunoglobulina humana

El reactivo más importante de la FCXM es el anticuerpo secundario contra la IgG humana, el cual se usa para identificar los anticuerpos específicos contra el donante que están específicamente unidos a las células blanco y, por ello, debe ser seleccionado para proporcionar óptimas sensibilidad y especificidad. Se pueden seleccionar anticuerpos secundarios específicos para las diversas clases y subclases de inmunoglobulinas. Un anticuerpo secundario óptimo debería mostrar las siguientes características:

1. tener una gran especificidad contra la IgG humana;
2. contener solo el fragmento de unión a antígeno (*Fragment AntigenBinding*, Fab) para reducir la unión inespecífica a los FcR, y
3. mostrar una mínima reactividad cruzada contra la IgM humana y las inmunoglobulinas de otras especies.

En la actualidad, el anticuerpo secundario más utilizado es un anticuerpo F(ab)2 policlonal contra la IgG humana (específico contra el Fc) conjugado con isotiocianato de fluoresceína. Cada lote de este anticuerpo secundario requiere una extensa validación y se debe determinar la concentración óptima antes de su uso. Para ello, se prueban varias diluciones del anticuerpo secundario contra los linfocitos T y B que han sido preincubadas con un suero de control positivo bien definido. Aunque se puede ver una mayor intensidad de fluorescencia con concentraciones mayores del anticuerpo secundario, con concentraciones más bajas se puede observar un valor más alto de la diferencia entre las MCS.

Esto es el resultado principal de una reactividad inespecífica menor del anticuerpo secundario en concentraciones más bajas del reactivo. Se ha observado una diferencia significativa en los niveles de reactividad inespecífica entre lotes de este tipo de anticuerpo secundario. Por consiguiente, es difícil encontrar dos lotes que tengan una sensibilidad y especificidad comparables. Por ello, los laboratorios de histocompatibilidad han comenzado a introducir anticuerpos monoclonales contra la IgG humana conjugados con isotiocianato de fluoresceína como anticuerpo secundario, con los cuales la variabilidad entre lotes es mínima ^10^^,^^11^, probablemente por la naturaleza monoclonal del anticuerpo, así como por el método de producción *in vitro*. Como se observa en el cuadro 3, una comparación de las características de rendimiento de la FCXM realizada con un anticuerpo policlonal frente a la realizada con un anticuerpo monoclonal, muestra que este último presenta una sensibilidad y una especificidad significativamente mayores ^10^^,^^11^.

**Cuadro 3:** Características de rendimiento de la prueba cruzada por citometría de flujo con el anticuerpo secundario policonal, comparada con la prueba cruzada por citometría de flujo con el anticuerpo secundario monoclonal

	FCXM de linfocitos T (n=100)		FCXM de linfocitos B (n=100)	
	Anticuerpo policlonal		Anticuerpo policlonal	
	Positivo	Negativo	Positivo	Negativo
Anticuerpo monoclonal				
Positivo	70	4	59	26
Negativo	2	24	1	14
Sensibilidad (IC_95%_)	94,6 % (86,7 - 98,5 %)		69,4 % (58,5 - 79 %)	
Especificidad (IC_95%_)	92,3 % (74,9 - 100 %)		93,3 % (69,0 - 99,8 %)	
VPP (IC_95%_)	97,2 % (90,3 - 99,7 %)		98,3 % (91,1 - 100 %)	
VPN (IC_95%_)	85,7 % (67,3 - 96 %)		35 % (20,6 - 51,7 %)	
Valor de p	<0,0001		<0,0001

FCXM: prueba cruzada por citometría de flujo; IC: intervalo de confianza; VPP: valor predictivo positivo; VPN: valor predictivo negativo

Estos datos indican que la mayor sensibilidad y especificidad observadas con el anticuerpo monoclonal se deben a una menor reactividad inespecífica observada con este tipo de anticuerpo ^71^^,^^72^. El problema de la reactividad cruzada contra las inmunoglobulinas de otras especies tampoco existe cuando se utiliza un anticuerpo monoclonal que, además, puede contribuir a su mejor rendimiento. El anticuerpo monoclonal es más costoso que el anticuerpo policlonal, pero la menor variabilidad entre los diferentes lotes y la sensibilidad y especificidad mayores, lo convierten en un reactivo óptimo para su uso en la FCXM.

## Controles positivos y negativos

La FCXM requiere un control positivo y un control negativo. Sin embargo, no existen controles comerciales específicamente diseñados para la prueba. Por lo tanto, cada laboratorio debe establecer sus propios reactivos de control y sus valores de corte positivos para su interpretación. El suero humano normal utilizado como control negativo es el reactivo de control más importante. Este reactivo se suele obtener de un grupo de donantes sanos sin anticuerpos anti-HLA detectados utilizando pruebas con microesferas de antígeno único.

Como alternativa, hay varios sueros humanos normales disponibles comercialmente compuestos por sueros de un gran número de donantes masculinos sanos con grupo sanguíneo AB, que pueden utilizarse como control negativo. No obstante, se debe hacer un examen minucioso de estos posibles controles negativos utilizando una FCXM con células de diferentes donantes (n≥5) y pruebas de anticuerpos anti-HLA con microesferas de antígeno único para garantizar que el reactivo no tenga una reactividad inespecífica contra los linfocitos y, más importante, que no tenga reactividad contra el HLA.

Para determinar el rango normal de intensidad de la fluorescencia obtenida con el control negativo o con sueros de pacientes sin anticuerpos anti-HLA, deben practicarse varias FCXM utilizando células de individuos cuyos tipos de HLA representen sus especificidades más comunes (n≥20). La intensidad de fluorescencia de cada FCXM debe registrarse sin cambiar la configuración del instrumento.

A continuación, deben calcularse la media y la desviación estándar de la intensidad de la fluorescencia de las poblaciones de linfocitos T y B. Utilizando un valor entre 2 y 3 desviaciones estándar (que cubre entre el 95,4 y el 99,7 % de los resultados), es posible establecer un valor de corte que puede utilizarse como indicador de positividad ^8^^,^^24^. Así, con un alto nivel de confianza estadística, si la intensidad de fluorescencia observada con el suero de un paciente es mayor que el valor de corte elegido, la muestra se considera positiva. La distinción entre los resultados débilmente positivos y los negativos es particularmente problemática en la FCXM. Por esta razón, cada laboratorio debe establecer sus valores indicadores de positividad. Sin embargo, estos no son absolutos y deben establecerse nuevos valores de corte con cada nuevo lote de control negativo.

A diferencia del control negativo, el control positivo es más fácil de obtener. Este reactivo suele consistir en un conjunto de sueros de pacientes con grandes concentraciones de anticuerpos anti-HLA, con un panel de anticuerpos reactivos igual o superior al 80 % y cuyo perfil de anticuerpos combinados cubra cerca del 100 % de los HLA. Un reactivo de control positivo no diluido normalmente produce resultados positivos muy altos que raramente se observan con el suero de un paciente. Por lo tanto, es importante utilizar el control positivo en una dilución que produzca una intensidad de fluorescencia moderada (a niveles no saturantes de los anticuerpos específicos contra el donante).

En este sentido es útil subrayar que la FCXM debe ser óptima para detectar pequeñas concentraciones de anticuerpos anti-HLA. Por lo tanto, el uso de un control positivo que presente una intensidad de fluorescencia moderada, ayudará en la evaluación diaria de su desempeño. Por ejemplo, si el control positivo muestra regularmente un MCS de 120 a 140, cuando el valor de corte para un resultado positivo es de 20 a 30 MCS (en la escala de 0-256 canales), entonces, una FCXM en la que el control positivo muestre un valor de MCS de 40 a 60 puede indicar que la prueba no es válida. Por el contrario, un control positivo muy fuerte, que normalmente arroje un MCS de 220 a 240, no añade ninguna información útil. En este caso, se pueden cometer errores importantes que no necesariamente dan lugar a un cambio significativo en el MCS del control positivo; en cambio, un control positivo con una intensidad de fluorescencia moderada es más sensible a las desviaciones técnicas del sistema.

## Comparación entre la prueba cruzada por citotoxicidad dependiente del complemento y la prueba cruzada por citometría de flujo

Actualmente, la mayoría de los centros de trasplantes consideran la FCXM como la prueba de referencia para la detección de anticuerpos específicos contra el donante. Indiscutiblemente es una técnica más sensible para su detección y un mejor predictor del resultado del trasplante renal que la CDCXM y la AHG-CDCXM ^12^^,^^13^^,^^17^^,^^19^^,^^21^^,^^22^^,^^26^^,^^29^^,^^73^. Sin embargo, hasta la fecha no se ha hecho ningún análisis directo para determinar la correlación entre el resultado de la CDCXM y la reactividad de la FCXM.

**Figura 2:**
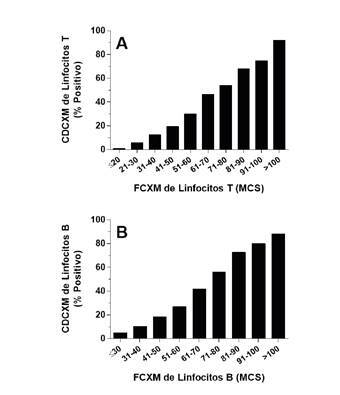
Comparación entre las pruebas cruzada de citotoxicidad dependiente del complemento y la prueba cruzada de citometría de flujo. La CDCXM y la FCXM de linfocitos T (n=30.303) (A) y de linfocitos B (n=27.686) (B), se realizaron en paralelo. A continuación, se comparó la reactividad de la FCXM y el resultado de la CDCXM mediante regresión lineal. La intensidad de la fluorescencia de la FCXM se capturó como datos lineales en la escala de 0-256 canales.

Para determinar la correlación entre una y otra, en nuestro laboratorio (Mayo Clinic, Phoenix, AZ, USA), hicimos un análisis retrospectivo de estas pruebas cruzadas practicadas simultáneamente ^10^^,^^11^. En el cuadro 4, se observa que el CDCXM de linfocitos T y B mostró una sensibilidad significativamente menor en comparación con la FCXM de linfocitos T y B (34,7 y 40 %, respectivamente). Además, estos resultados demostraron una correlación lineal entre el resultado de la CDCXM y la reactividad de la FCXM (figuras 2A-B). Es interesante que menos del 50 % de las CDCXM fueron positivos cuando la FCXM evidenciaba un MCS inferior a 70-80 (en la escala de 0-256 canales). Incluso con MCS superiores a 100 en esa misma escala, el porcentaje de resultados positivos del CDCXM de linfocitos T y B solo alcanzó el 92 y el 88 %, respectivamente. Estos datos demuestran la sensibilidad significativamente menor de la CDCXM en comparación con la FCXM, y la capacidad de esta para detectar anticuerpos que no activan el complemento.

**Cuadro 4:** Características de rendimiento de la prueba cruzada por citotoxicidad dependiente del complemento, comparada con la prueba cruzada por citometría de flujo

	Linfocitos T (n=30.303)		Linfocitos B (n=27.686)	
	CDCXM		CDCXM	
	Positivo	Negativo	Positivo	Negativo
FCXM				
Positivo	2.968	5.580	3.297	4.939
Negativo	217	21.538	988	18.462
Sensibilidad (IC_95%_)	34,7 % (33,7 - 35,7 %)		40 % (39 - 41,1 %)	
Especificidad (IC_95%_)	99 % (98,9 - 99,1 %)		94,9 % (94,6 - 95,2 %)	
VPP (IC_95%_)	93,2 % (92,3 - 94 %)		76,9 % (75,7 - 78,2 %)	
VPN (IC_95%_)	79,4 % (78,9 - 79,9 %)		78,9 % (78,4 - 79,4 %)	
Valor de p	<0,0001		<0,0001

CDCXM: prueba cruzada por citotoxicidad dependiente del complemento; FCXM: prueba cruzada por citometría de flujo; IC: intervalo de confianza; VPP: valor predictivo positivo; VPN: valor predictivo negativo

## Prueba cruzada virtual

El concepto de la prueba cruzada virtual (*Virtual Crossmatch*, VXM) ha sido debatido por la comunidad de trasplantes durante muchos años ^74^^,^^75^. La VXM es el proceso de evaluación de los resultados de las pruebas de anticuerpos anti-HLA para predecir los resultados de la prueba cruzada física.

A pesar de su nombre, la VXM no es una verdadera prueba cruzada en el sentido de enfrentar las células del donante y el suero del receptor potencial, más bien, es un análisis en paralelo del perfil de anticuerpos anti-HLA del paciente y de la tipificación del HLA del donante para predecir el resultado de la prueba cruzada física. En varios estudios se ha demostrado que una VXM negativa (ausencia de anticuerpos específicos contra el donante) representa un riesgo muy bajo de rechazo mediado por anticuerpos y una buena supervivencia del injerto. Por el contrario, una VXM positiva (presencia de anticuerpos específicos contra el donante) representa un riesgo significativo de rechazo mediado por anticuerpos y una disminución de la supervivencia del injerto ^75^^-^^88^.

Otra aplicación de la VXM es su uso para la asignación de órganos. En diversos estudios se ha demostrado que la VXM permite, cuando no es viable una prueba cruzada física, importar órganos provenientes de fuera de la zona local de distribución de órganos para pacientes muy sensibilizados, permitiendo, así, una rápida asignación de órganos con una segura evaluación de la histocompatibilidad inmunológica entre el receptor potencial y el donante antes del trasplante.

Esta estrategia aumenta considerablemente el acceso a órganos inmunológicamente compatibles para pacientes muy sensibilizados con excelentes resultados a largo plazo ^75^^-^^88^. Por consiguiente, la VXM ofrece la oportunidad de ampliar el área geográfica en la cual se pueden recuperar órganos con un tiempo de isquemia fría permisible y reduce la tasa de mortalidad en la lista de espera, además de ser un excelente indicador de riesgo del rechazo mediado por anticuerpos.

Las limitaciones de la VXM deben considerarse cuidadosamente, ya que las concentraciones de anticuerpos específicos contra el donante pueden variar significativamente con el tiempo. Por lo tanto, un análisis del perfil de estos anticuerpos de una muestra “histórica” con más de seis meses de antigüedad, no puede predecir con certeza el resultado de una prueba cruzada física con una muestra actual. Los eventos de sensibilización, como transfusiones de sangre, trasplantes o embarazos, pueden cambiar sustancialmente el perfil de los anticuerpos específicos actuales comparados con la muestra “histórica”.

Los elementos críticos de una VXM son la evaluación precisa del perfil de anticuerpos específicos contra el donante actuales y el análisis de los factores que pueden influir en el resultado de la prueba cruzada física ^20^^,^^33^^-^^38^. Por lo tanto, la VXM debe realizarse considerando todos los resultados disponibles de los anticuerpos específicos contra el donante, incluyendo por lo menos una muestra reciente (de menos de tres meses de antigüedad). Además, la VXM puede dar un falso positivo en el caso de niveles bajos de múltiples anticuerpos específicos contra el donante, lo que excluiría erróneamente a donantes inmunológicamente compatibles.

Análogamente, los pacientes pueden presentar anticuerpos específicos contra el donante específicos de alelo (por ejemplo, anticuerpos contra HLA-A*02:01 pero no contra HLA-A*02:03), lo que puede excluir equivocadamente a donantes con HLA-A*02:03, ya que rara vez se dispone de tipificación de alta resolución para donantes fallecidos ^89^. Alternativamente, la VXM puede ser un falso negativo, ya que la lista creciente de alelos de HLA no queda representada completamente en las pruebas de anticuerpos anti-HLA. Aunque esto es teóricamente posible, es poco común porque las pruebas de anticuerpos anti-HLA actuales incluyen los alelos de HLA más comunes definidos serológicamente. Se debe tener cuidado para garantizar que los alelos del donante estén representados en las pruebas de anticuerpos anti-HLA para reportar una VXM como negativa.

La correlación entre la VXM y la prueba cruzada física, es muy variable y depende de los métodos y criterios utilizados en cada laboratorio. Hasta que se logre una mejor estandarización, el enfoque aceptado es que también se debe realizar una prueba cruzada física, ya sea prospectiva o retrospectivamente, dependiendo de las políticas internas de cada programa de trasplantes.

La determinación de los niveles de anticuerpos específicos contra el donante que se consideran admisibles para el trasplante en función de la correlación entre las pruebas de anticuerpos anti-HLA y los resultados de las pruebas cruzadas, disminuye el número de resultados positivos inesperados de la prueba cruzada física, con lo que se reduce el trabajo innecesario en el laboratorio ^88^^,^^90^^,^^91^. La implementación de la VXM en la asignación de órganos depende de una evaluación precisa de las concentraciones de anticuerpos específicos contra el donante, ya que se correlaciona con el riesgo de rechazo mediado por anticuerpos.

Como se ha mencionado anteriormente, hay varios estudios sobre la viabilidad y la utilidad de la VXM ^75^^-^^88^. También, se han hecho otros para investigar su valor predictivo frente al CDCXM y la FCXM ^49^^,^^83^^,^^87^^,^^88^^,^^92^^-^^94^. La correlación de la VXM con la CDCXM es muy baja, pero esto no es sorprendente dada la mayor sensibilidad de las pruebas en fase sólida de anticuerpos anti- HLA ^87^^,^^88^. Por el contrario, la correlación de la VXM con la FCXM ha sido superior al 85 % en la mayoría de los estudios ^74^^,^^75^^,^^79^^,^^87^^,^^93^^,^^94^.

Cabe señalar que Zachary, *et al*., demostraron que el nivel de anticuerpos específicos contra el donante definido por pruebas de anticuerpos anti-HLA con microesferas de fenotipo, se correlacionan significativamente con el resultado tanto de la CDCXM como de la FCXM ^88^. Los autores pudieron predecir los resultados de la CDCXM y de la FCXM en el 92,8 y el 92,4 % de los casos, respectivamente. Es importante señalar que las correlaciones con las pruebas de anticuerpos anti-HLA con microesferas de antígeno único fueron sustancialmente menores (82,6 y 47,9 %, respectivamente) y pueden deberse a varios factores, entre ellos, la gran variabilidad de la concentración de los diferentes alelos de HLA en las microesferas y la sensibilidad significativamente más alta de las pruebas de anticuerpos anti-HLA con microesferas de antígeno único ^88^.

En un estudio de nuestro laboratorio (Mayo Clinic, Phoenix, AZ, USA), se hizo un análisis de la correlación entre los niveles de anticuerpos específicos contra el donante medidos por pruebas de anticuerpos anti- HLA con microesferas de antígeno único y la reactividad de la FCXM. Debe mencionarse que, en los casos con múltiples anticuerpos específicos contra el donante, se utilizaron los valores acumulados de la intensidad media de la fluorescencia para el análisis ^10^. Además, los pacientes con anticuerpos contra los epítopos Bw4 o Bw6 fueron excluidos del estudio ^95^^,^^96^ porque estos epítopos mutuamente excluyentes son compartidos por múltiples HLA y, por lo tanto, los anticuerpos complementarios contra Bw4 o Bw6 se reparten en un gran número de microesferas en las pruebas de anticuerpos anti-HLA. En consecuencia, hay menos anticuerpos uniéndose a una sola microesfera, lo que subestima significativamente el nivel de anticuerpos específicos contra el donante contra Bw4 o Bw6 ^97^^,^^98^.

**Figura 3:**
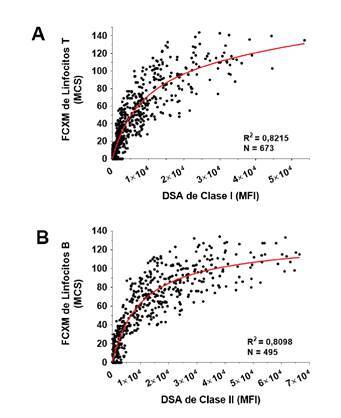
Comparación entre las pruebas de anticuerpos anti- HLA con microesferas de antígeno único y la prueba cruzada por citometría de flujo. La FCXM de linfocitos T (A) y la de linfocitos B (B), se hicieron en paralelo con pruebas de anticuerpos anti-HLA utilizando microesferas de antígeno único. Se comparó la reactividad de la FCXM de linfocitos T y los niveles de anticuerpos especificos contra el donante de clase I (HLA-A, B, C) (A) y la reactividad de la FCXM de linfocitos B y los niveles de anticuerpos especificos contra el donante de clase II (HLA-DRB1, DRB3/B4/B5, DQB1, DPB1) (B). En los casos con múltiples anticuerpos especificos contra el donante, se utilizaron en el análisis los valores de intensidad media de fluorescencia (*Mean Fluorescence Intensity*, MFI) acumulados. Los pacientes con anticuerpos contra los epítopos públicos Bw4 o Bw6 se excluyeron de este estudio. La correlación entre los valores de MCS y MFI se analizaron mediante la prueba de regresión lineal. La intensidad de la fluorescencia de la FCXM se capturó como datos lineales en la escala de 0-256 canales.

Se demostró, asimismo, una correlación significativa entre los niveles de anticuerpos específicos contra el donante de clase I (HLA-A, B, C) y la reactividad de la FCXM de linfocitos T (figura 3a), así como una correlación significativa similar entre los niveles de anticuerpos específicos contra el donante de clase II (HLA-DRB1, DRB3/B4/B5, DQB1, DPB1) y la reactividad de la FCXM de linfocitos B (figura 3B).

En general, estos datos demuestran que la VXM es una herramienta precisa y útil para predecir el resultado de la prueba cruzada física. Aunque una concordancia del 100 % es deseable, ambas pruebas tienen sus limitaciones inherentes, lo que hace que una correlación perfecta sea muy difícil de alcanzar. Es importante destacar que tanto la predicción de la prueba cruzada física como la evaluación del riesgo de rechazo mediado por anticuerpos, exigen conocer las deficiencias de las pruebas de anticuerpos anti-HLA, un estricto control de calidad del análisis de los datos y una evaluación continua de la correlación entre las pruebas de anticuerpos anti- HLA y los resultados de la prueba cruzada física.

## Prueba cruzada por citometría de imagen

Ciertamente, el citómetro de flujo sigue siendo un excelente instrumento para una prueba relativamente simple como la FCXM. Entre sus beneficios, se pueden destacar la alta sensibilidad y especificidad, la detección simultánea de múltiples analitos y más de 30 años de experiencia en el laboratorio clínico.

Por otro lado, el alto costo de este instrumento, su costoso mantenimiento, compleja calibración y exigente operación, representan las limitaciones fundamentales para los laboratorios de histocompatibilidad que manejan poco volumen de pruebas.

Sin embargo, desde la primera descripción de la FCXM en 1983 ^27^, los avances tecnológicos en la adquisición de imágenes digitales, microfluidos y diodos emisores de luz como fuente de luz de excitación de fluorocromos, han permitido el desarrollo de instrumentos simples y compactos capaces de reproducir algunas de las funciones del citómetro de flujo. La sustitución de los rayos láser por diodos emisores de luz y de complejos sistemas de fluidos por cámaras de microfluidos compactas, ha dado lugar a instrumentos pequeños, menos costosos y que prácticamente no necesitan inversión en mantenimiento.

En un estudio realizado en nuestro laboratorio (Mayo Clinic, Phoenix, AZ, USA), se exploró la posibilidad de emplear un citómetro de imagen, el Cellometer Vision CBA™ (Nexcelom Bioscience LLC), como instrumento alternativo para la prueba cruzada ^99^. Los resultados de la prueba cruzada por citometría de imagen (*Image Cytometry Crossmatch*, IXM) tuvieron una concordancia del 96 % cuando esta se comparó con la FCXM.

**Figura 4:**
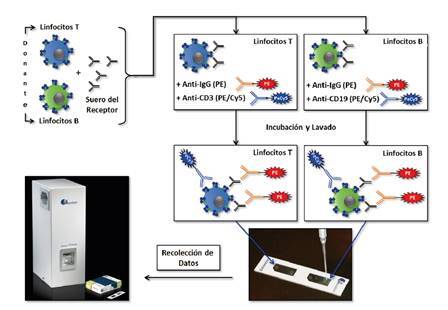
Protocolo de la prueba cruzada por citometría de imagen de dos colores. El protocolo de la IXM de dos colores se adaptó del protocolo de la FCXM de tres colores.

Los resultados de este estudio demostraron que la IXM de dos colores adaptada de la FCXM de tres colores, podría ser analizada e interpretada con éxito en el citómetro de imagen (figura 4). Los resultados de las imágenes fluorescentes captadas por el citómetro de imagen y su posterior análisis permitieron la correcta caracterización de las poblaciones de linfocitos T y B en las proporciones esperadas en sujetos sanos.

**Figura 5:**
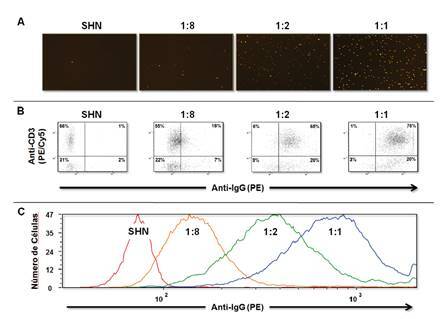
Análisis de diluciones de la prueba cruzada por citometría de imagen. La intensidad de los eventos capturados en la imagen digital por módulos de PE de un control positivo en diferentes diluciones (1:1, 1:2, 1:8) y de un suero humano normal como control negativo (A). Al igual que en la FCXM, la intensidad de los eventos se muestra en gráficos de dispersión (B). Los desplazamientos de la población linfoide T se representan en un gráfico superpuesto de un control positivo en diferentes diluciones (1:1, 1:2, 1:8) y de un suero humano normal como control negativo (C).

En un experimento de titulación con una muestra que contenía anticuerpos específicos contra el donante de clase I y de clase II, se demostró que el sistema es capaz de detectar diferentes concentraciones de estos anticuerpos que reaccionan con los linfocitos T y B, respectivamente (figura 5). Estos resultados también demostraron que la linealidad y la sensibilidad de ambos instrumentos son comparables. El estudio paralelo de 39 pruebas cruzadas de IXM utilizando la FCXM como método de referencia, demostró una excelente sensibilidad y especificidad (cuadro 5). Se observaron tres resultados discrepantes, uno para la prueba cruzada de linfocitos T y dos para la prueba cruzada de linfocitos B. En todos estos casos, la IXM mostró una mejor correlación con la VXM.

**Cuadro 5:** Características de rendimiento de la prueba cruzada por citometría de imagen comparada con la prueba cruzada por citometría de flujo

	Linfocitos T (n=39)		Linfocitos B (n=39)	
	IXM		IXM	
	Positivo	Negativo	Positivo	Negativo
FCXM				
Positivo	16	1	21	0
Negativo	0	22	2	16
Sensibilidad (IC_95%_)	94,1 % (71,3 - 99,9 %)		100 % (83,9 - 100 %)	
Especificidad (IC_95%_)	100 % (84,6 - 100 %)		88,9 % (65,3 - 98,6 %)	
VPP (IC_95%_)	100 % (79,4 - 100 %)		91,3 % (72 - 98,9 %)	
VPN (IC_95%_)	95,7 % (78,1 - 99,9 %)		100 % (79,4 - 100 %)	
Valor de p	<0,001		<0,001

IXM: prueba cruzada por citometría de imagen; FCXM: prueba cruzada por citometría de flujo; IC: intervalo de confianza; VPP: valor predictivo positivo; VPN: valor predictivo negativo

La utilidad clínica de la detección óptica de anticuerpos anti-HLA mediante técnicas inmunofluorescentes ya fue evaluada ^100^, mediante una prueba cruzada basada en la inmunofluorescencia indirecta examinada por microscopía de fluorescencia con contraste de fase. Lobo, et al., demostraron una sensibilidad significativamente mayor que la de la CDCXM y una mejor correlación clínica con el rechazo acelerado ^100^. Estos resultados, y los obtenidos con el citómetro de imagen ya descritos, permitieron estimar que la IXM tiene una utilidad clínica comparable a la de la FCXM, lo que le confiere el potencial de ser utilizada en el ámbito clínico para la selección de donantes y la evaluación del riesgo de rechazo mediado por anticuerpos.

Además de la concordancia entre los resultados de la IXM y la FCXM, el método de citometría de imagen tiene varias ventajas técnicas sobre la citometría de flujo. Utilizando la combinación de marcadores fluorescentes como la ficoeritrina y la ficoeritrina/cianina 5 sin superposición espectral de emisiones, se generaron imágenes fluorescentes para ambos canales sin interferencia óptica y sin necesidad de una extensa optimización de filtros. Además, la ausencia de rayos láser o de tubos fotomultiplicadores en el citómetro de imagen elimina la necesidad de una alineación óptica precisa, además de que la sencilla configuración de la epifluorescencia no requiere mantenimiento diario.

Por último, debido a la sencillez de la arquitectura óptica, el citómetro de imagen no tiene un complejo sistema fluídico para calibrar o mantener. Los citómetros de flujo pequeños pueden ser económicamente accesibles, pero aun así, hay la posibilidad de obstrucción del sistema fluídico, lo que pone en riesgo la integridad de la muestra. Debido a la ausencia de un complejo sistema fluídico para cebar, se requiere menor cantidad de muestra. En nuestro estudio, se demostró la capacidad y la fiabilidad del citómetro de imagen como un instrumento alternativo para la prueba cruzada. La fácil operación sin costos ni esfuerzos adicionales de calibración diaria, así como su menor costo de adquisición, hacen de este instrumento una excelente alternativa para los laboratorios de histocompatibilidad con bajo volumen.

## Conclusión

La prueba cruzada es actualmente la única prueba de histocompatibilidad que se hace en la mayoría de los laboratorios. La CDCXM y la AHG- CDCXM son útiles para detectar la mayoría de los anticuerpos específicos contra el donante responsables del rechazo hiperagudo, pero carecen de la sensibilidad suficiente para proteger adecuadamente contra otros tipos de rechazo mediado por anticuerpos.

Los avances en la citometría de flujo, la biología molecular y la química de proteínas, contribuyeron al desarrollo de la FCXM, la cual proporciona un método más sensible para evaluar la histocompatibilidad inmunológica entre el receptor potencial y el donante, antes del trasplante. Conjuntamente con la pruebas en fase sólida de anticuerpos anti-HLA que permiten una VXM muy precisa, la prueba cruzada ha contribuido significativamente a nuestra comprensión del papel de los anticuerpos específicos contra el donante en el proceso de rechazo del injerto y ha facilitado un gran número de trasplantes en pacientes muy sensibilizados con un gran margen de seguridad.
